# Dual-Band Perfect Metamaterial Absorber Based on an Asymmetric H-Shaped Structure for Terahertz Waves

**DOI:** 10.3390/ma11112193

**Published:** 2018-11-06

**Authors:** Taiguo Lu, Dawei Zhang, Peizhen Qiu, Jiqing Lian, Ming Jing, Binbin Yu, Jing Wen, Songlin Zhuang

**Affiliations:** 1 Engineering Research Center of Optical Instrument and System, Ministry of Education and Shanghai Key Laboratory of Modern Optical System, University of Shanghai for Science and Technology, Shanghai 200093, China; lvtaiguo@lcu.edu.cn (T.L.); qiupeizhen@126.com (P.Q.); lianjiqing1990@163.com (J.L.); jingming_usst@163.com (M.J.); pai3_14@yeah.net (B.Y.); jwen@usst.edu.cn (J.W.); slzhuang@yahoo.com (S.Z.); 2 School of Physical Science and Information Engineering, Liaocheng University, Liaocheng 252059, China; 3 Department of Applied Physics, Huzhou University, Huzhou 313000, China

**Keywords:** metamaterial absorber, H-shaped structure, asymmetry, multi-reflection interference

## Abstract

We designed an ultra-thin dual-band metamaterial absorber by adjusting the side strips’ length of an H-shaped unit cell in the opposite direction to break the structural symmetry. The dual absorption peaks approximately 99.95% and 99.91% near the central resonance frequency of 4.72 THz and 5.0 THz were obtained, respectively. Meanwhile, a plasmon-induced transmission (PIT) like reflection window appears between the two absorption frequencies. In addition to theoretical explanations qualitatively, a multi-reflection interference theory is also investigated to prove the simulation results quantitatively. This work provides a way to obtain perfect dual-band absorption through an asymmetric metamaterial structure, and it may achieve potential applications in a variety of fields including filters, sensors, and some other functional metamaterial devices.

## 1. Introduction 

Over the past decades, the development of the terahertz (THz) technique was slow because of a shortage of functional devices, but which attracted much attention due to their many potential applications, such as communications, security, and biotechnology [1,2,3]. Since Landy et al. [4] proposed a metamaterial absorber to obtain near unity absorption, more and more THz functional devices based on metamaterial have been made, such as absorbers, filters, sensors, and emitters [5,6,7,8]. In recent years, graphene with special properties has been studied and used to fabricate absorbers because it is connected with surface plasmon polaritons (SPPs) in the THz region much like the noble metals [9,10]. One of the graphene applications involves designing tunable THz metamaterial because its sheet conductivity can be continuously tuned [11]. The structure of metal nanoparticles on polymer also exhibits excellent performance in shielding and absorption. The nanoparticles will interact with other nanoparticles through the evanescent field and enhance their near electromagnetic field when they are illuminated [12].

Metamaterials, first proposed by Veselagoin [13] in 1968, are artificially engineered materials which are attractive not only for their ability to induce strong electric and magnetic responses through the interaction between the incident electromagnetic wave and the designed metamaterial pattern with special shape and size, but also for their potential extensive applications, including electromagnetic wave absorber, which has been a research hotspot due to their ability to absorb incident waves with near unity absorption. Hu Tao [14] designed and fabricated the first THz narrow-band absorber. Thereafter, dual band absorbers, multiband absorbers, frequency tunable absorbers, and broadband absorbers were also reported [15,16,17,18]. The exciting advantage is that the absorption properties of metamaterials are determined mainly by the size and shape of element unit cells rather than their composition, for example the common structure cut wire [19], split-ring [20], U-shaped structure [21], F-Shaped structure [22], etc. In the past, symmetric structures were mainly used to obtain high absorption, but now some deformations of symmetric structures are used to achieve perfect absorption at THz frequency [23,24]. Some fascinating phenomena appear when the symmetry of the structure is broken, such as multi-resonance that is suitable for developing multi-band or broadband absorption [25]. The H-shaped symmetric or asymmetric structure is one of the most used elements to design metamaterial devices [26,27,28,29,30]. 

In our work, contrasting with H-shaped structure mentioned above, we designed a simple ultra-thin asymmetric structure by adjusting the side strips’ length of the H-shaped unit cell in the opposite direction simultaneously. Through the asymmetric H-shaped metamaterial structure, we obtained dual band absorption peaks with absorptivity of about 99.95% and 99.91% at the resonance frequency 4.72 THz and 5.0 THz, respectively. Meanwhile, a PIT -like reflection window appeared between the absorption frequency. While the H-shaped structure was in symmetry case, there was only one obvious narrow-band absorption peak at 4.28 THz. We found that the symmetry of the H-shaped structure was broken in the x-axis or y-axis direction alone; the absorption peak at low absorption frequency was quite different from that at high frequency. In addition to qualitative descriptions, we prove the results using the multi-reflection interference theory quantificationally. The absorption spectra and the central frequency of the reflection window as a function of the asymmetric H-shaped unit cell parameters for the various Δx, width of the central strip, and the thickness of the dielectric layer was observed with inversely proportional relation.

The metamaterial structure was ultra-thin with the thickness no more than 8μm, and we could obtain dual band perfect absorption. We believe that this design concept can be applied for sensors, filters, diagnostics, telecommunications, etc. For example, THz radar is attracting the eyes of many researchers in military fields in recent years; the asymmetric metamaterial formed on the surface of THz radar by machining methods promises to be one of the cloaking materials in anti-reconnaissance.

## 2. Design and Results

It was considered that there are interesting phenomena if the symmetry structure is broken, and the asymmetry is considered to be a prerequisite for the PIT-like reflection window [31,32]. We believe we can find some different phenomena through breaking the symmetric H-shaped structure.

**Figure 1:**
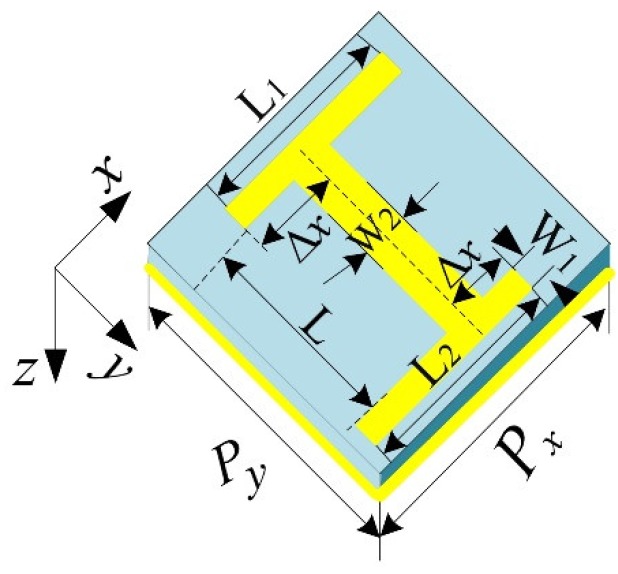
Schematic structure of an asymmetric H-shaped unit cell of the metamaterial. The geometric parameters of the unit cell are:Δx = 9 μm,L=28 μm, $L_{1}=L_{2}=27$ μm, $W_{1}=4$ μm, $W_{2}=5$ μm, $P_{x}=P_{y}=40$ μm.

**Figure 2:**
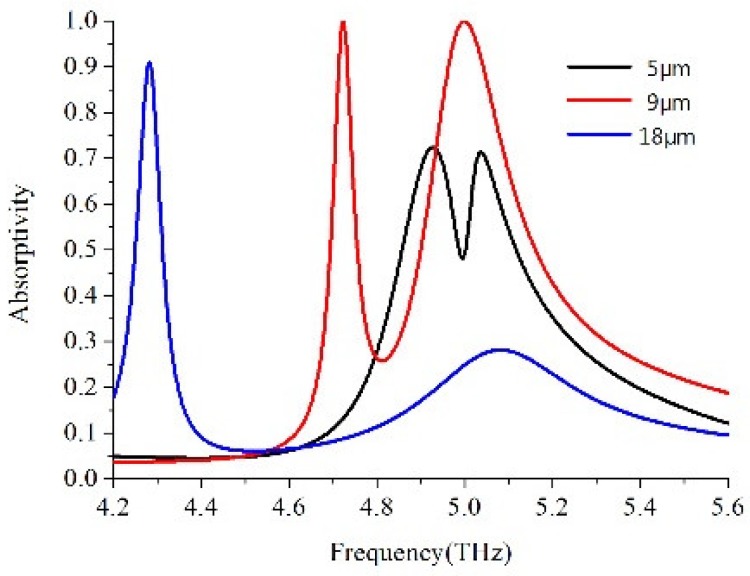
Simulated absorptivity for the symmetric H-shaped unit cell with Δx = 18 μm and asymmetric H-shaped unit cell with Δx = 5 and 9 μm.

The ultra-thin dual-band perfect absorber based on the metamaterial asymmetric H-shaped structure was designed and its properties were simulated and proven. The schematic of the asymmetric H-shaped unit cell is shown in Figure 1, which is formed by three layers: a continuous metallic plane layer on the bottom, a middle dielectric layer, and finally a second metallic layer with asymmetric H-shaped unit cells by varying the side strips’ length in the opposite direction periodically arranged on the top. Both of the top and bottom metallic layers are made of copper with a conductivity of 4.58 × 10^7^ S/m and an optimized thickness of 0.036 μm. The dielectric was FR-4 in the middle layer with an optimized thickness of 7 μm. The real permittivity assumed constant within the frequency range was 4.3 and a loss tangent was 0.025. When variable Δx = 9 μm, the geometry parameters of the asymmetric H-shaped unit cell were as follows: L=28 μm, $L_{1}=L_{2}=27$ μm, $W_{1}=4$ μm and $W_{2}=5$ μm. The variable Δx stands for the degree of the asymmetry. The periodicity of the H-shaped unit cell was fixed at 40 μm (square type) in the x-axis and y-axis directions. All the designs were performed utilizing the software CST Microwave Studio, choosing periodic boundary conditions along the x-axis and y-axis directions and open-add space condition along the z-axis direction. We now discuss the influence of the symmetry H-shaped structure breaking on absorption by changing Δx. A THz wave is supposed to be incidental perpendicularly onto the metamaterial surface, with its E polarization and H polarization along the y and x directions, respectively. The absorptivity A can be expressed as A = 1 – T − R, where T and R are transmission and reflection coefficients, respectively. However, the transmission is zero because of total reflection from the bottom metal layer, and the absorptivity expression can be simplified as A = 1 − R. Therefore, the absorptivity was determined by the reflectance alone, in order to achieve the minimum reflectance, the impedance-matching technique can be used to optimize the impedance of the metamaterial absorber [33]. In Figure 2, we can see there appears only a single obvious absorption peak with an absorptivity of 90.95% at a frequency of 4.28 THz and no apparent absorption peak in the symmetry case with Δx = 18 μm. When Δx = 9 μm, the symmetric H-shaped structure was broken in the x-axis and y-axis directions simultaneously, the previous single absorption peak is split into two absorption peaks with absorptivity of 99.95% and 99.91% at 4.72 THz and 5.0 THz, respectively. Meanwhile, a PIT-like reflection window with reflectivity of 74.23% appears between the two absorption peaks. When Δx=5 μm, the absorptivity and reflectivity were lower than that at Δx=9 μm. It can be found that the resonance was suppressed at the high frequency when the H-shaped structure was in the symmetry case; Once the symmetry was broken in the x-axis and y-axis directions simultaneously, the resonance at the higher frequency could be excited through plasmon near the field coupling. The magnitude of the absorption peak depends on the strength of the coupling [34].

The approach calculating the reflection coefficient is similar to that in Reference [35], but there are some differences essentially. The reflection coefficient in our work was calculated from superposition of multiple reflections, while the reflection coefficient in Reference [35] is calculated based on impedance obtained from the Transmission Line Theory. The way to calculate reflection coefficient in Reference [35] is accurate and efficient, but our way is more simple. The proposed device in Reference [35] realizes multi-band and broadband absorption in a wide range of angle of incidence, while there was dual band perfect absorption at normal incidence and PIT-like reflection window between the absorption frequency in our designed device and the absorptivity at the resonance frequency was higher than that in Reference [36].

**Figure 3:**
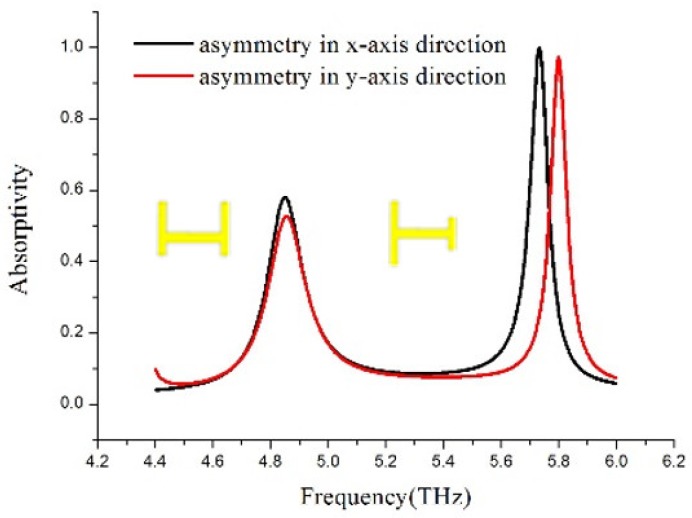
Absorption spectra for asymmetry in the H-shaped unit cell in the x-axis or in y-axis direction alone by changing the strips’ length with Δx = 9 μm in the same directions depicted as the inset.

Next, as a comparison with the above results, the absorption spectra was obtained when the symmetry of the H-shaped unit cell was broken partially in the x-axis or in the y-axis directions alone with Δx = 9 μm. As Figure 3 shows, the absorption peak at high frequency is a large difference to that at low frequency no matter the asymmetry in the x-axis or in the y-axis directions by changing the strips’ length with Δx = 9 μm in the same directions as the insets of Figure 3.

**Figure 4:**
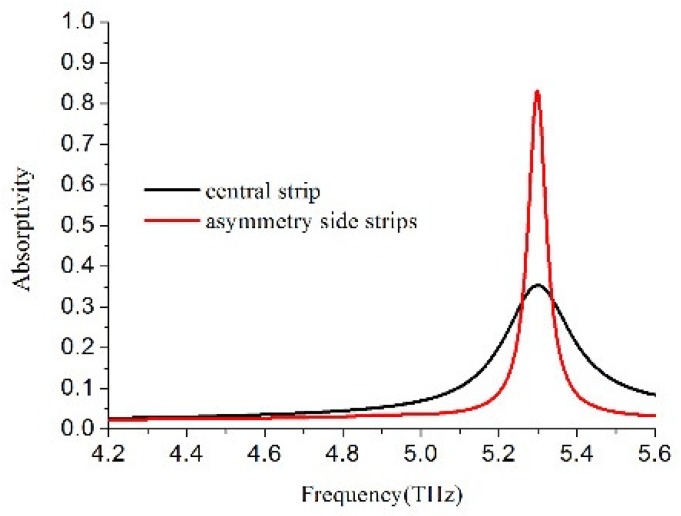
Individual absorption resonances of the central strip and asymmetrical side strips with the same resonance frequency, different Q factor, and different absorption strength.

**Figure 5:**
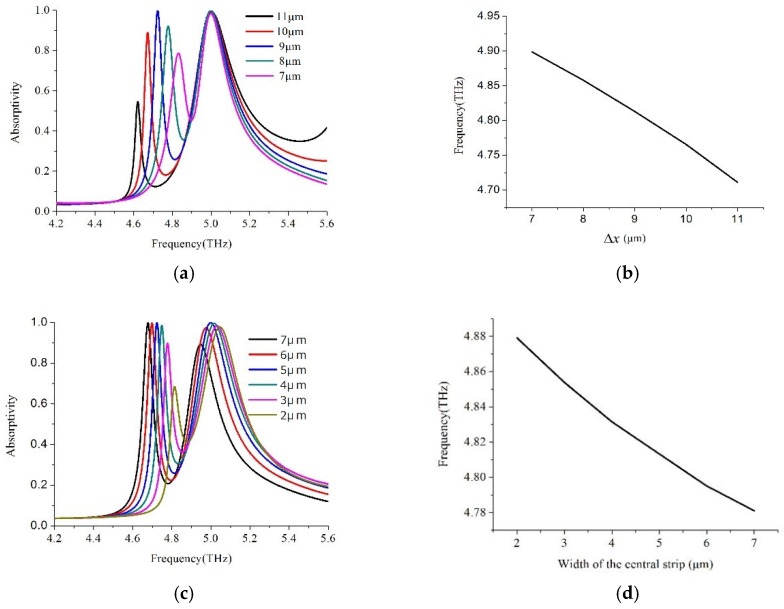

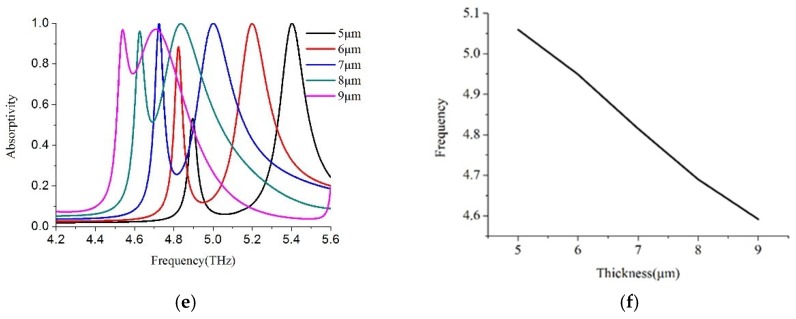
Simulated absorptivity as a function of the frequency for the (**a**) Δx and (**c**) width of the central strip, and (**e**) thickness of the FR-4 spacer. The central reflection frequency as a function of the frequency for the (**b**) Δx and (**d**) width of the central strip, and (**f**) thickness of the FR-4 spacer.

To investigate the physical mechanism for the production of the dual band absorption peaks and PIT-like reflection window, the whole H-shaped structure can be decomposed into two separate resonators: central strip resonator and a two-side strip resonator. In Figure 4, the individual central strip resonator and two-side strip resonator exhibit their respective absorption spectra. The resonance frequency for the central strip resonator was 5.3 THz with the absorptivity 35.35% and the quality factors 22 (the Q factor refers to the ratio of the center frequency to the full width at half maximum of a resonance), while the resonance frequency for the side strip resonator was also 5.3 THz with the absorptivity 83.13% and the quality factors (Q) 90. We can see that every resonator can provide one resonance mode with identical resonance frequency, but significantly different absorption strength and quality factors through comparative analyses, as described in References [37,38,39,40], there were two different excitation pathways of the resonance modes. As a composite structure consisting of the central strip and the side asymmetric strips, the resonance suppressed in the symmetric case was induced when the symmetry was broken. As a result, the primitive mode was split into two new modes with discrete energy levels through the near-field interaction and coupling. The influence of the asymmetric H-shaped structure parameters varying on absorptivity and reflectivity was elaborated. Figure 5a shows the simulated absorptivity spectra with absorptivity as a function of the frequency for the different Δx. We can see the low frequency has larger red shifting with the parameter Δx increase, but the high frequency is almost unaffected. The central reflection frequency as a function for the parameter Δx is plotted in Figure 5b; the central reflection frequency is inversely proportional to the parameter Δx. Figure 5c shows the simulated absorptivity spectra at different widths of the central strip. Both of the low and high resonance frequencies have red shifting when the width of the central strip increases. Figure 5d shows the central reflection frequency is inversely proportional to the width of the central strip. 

To investigate the effect of the thickness of the FR-4 dielectric layer, we calculated the absorptivity varying its thickness from 5 μm to 9 μm, as shown in Figure 5e,f. From Figure 5e, we can see that there are red shifts for the absorption peak at high frequencies and low frequencies with the increase of dielectric thickness, which leads to red shift of the reflection window. The central reflection frequency as a function of dielectric thickness is shown in Figure 5f, the central reflection frequency is inversely proportional to the dielectric thickness. The modulation of the frequency characteristic can be used to manufacture THz modulators and frequency selectors.

**Figure 6:**
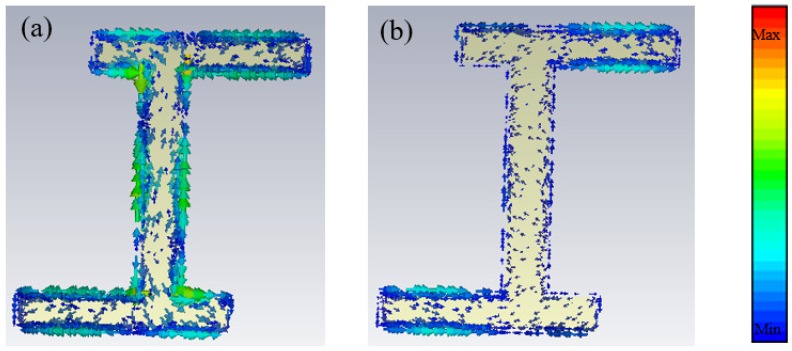
The distribution of induced currents at (**a**) low and (**b**) high resonant frequency.

We simulated the induced current distributions in the asymmetric H-shaped resonator at low- and high-frequency responses in order to elaborate the physical mechanism. As shown in Figure 6a, we can see there are circulating currents associated with the inductor-capacitor (LC) resonance and linear currents associating with the dipole resonance at low-resonance frequency. The incident electric field was strongly coupled with the top metallic patch and inducing currents; the currents in the resonator were out of phase causing destructive interference of the radiated fields and the incident energy was stored in this mode resulting in a high Q resonance. While in Figure 6b, the currents in the resonators were in phase causing constructive interference of the radiated fields and the incident energy was evaporated in this mode resulting in a low Q resonance. The modulation of the structure parameters on absorption spectra can be interpreted, the varying of Δx and the central strip width affected the LC resonance. When Δx and the central strip width increase, it will lead to an increase of the capacitance and inductance, and the resonance frequency will decrease according to Formula (1):

(1) $$f=\frac{1}{2\pi \sqrt{LC}}$$

When the spacer thickness increases, according to Reference [41], the absorption frequencies show continuous red shift.

## 3. Interference Theory

**Figure 7:**
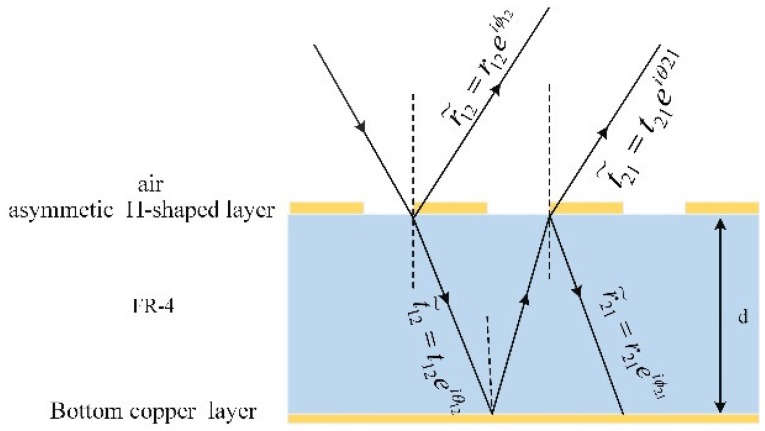
Multiple reflection and interference theory model of the metamaterial absorber.

The results were analyzed and explained qualitatively, and we will use interference theory to illustrate the correctness of the results quantitatively [42]. Figure 7 shows the multiple reflection and interference theory model of the proposed perfect THz absorber. For simplicity to discuss, we designed the model as two effective interfaces: the top air-dielectric interface and the bottom dielectric-backplane one. The thickness of the copper layers on the top and bottom are neglected without unchanging their function. In this model, the reflection/transmission coefficients of the metamaterial resonator and the phase change caused by the thickness of the dielectric spacer at the different layers should be obtained from simulations in order to calculate the overall reflection.

Suppose the THz wave irradiates perpendicularly on the surface from the air, the incident wave will be reflected partly back to the air at the top air-dielectric interface with a reflection coefficient ${\tilde{r}}_{12}=r_{12}e^{i\varphi_{12}}$ and the rest will be transmitted into the FR-4 spacer with a transmission coefficient ${\tilde{t}}_{12}=t_{12}e^{i\theta_{12}}$, after reaching to the bottom copper layer and then is totally reflected back to the top interface with a complex propagation phase β.The reflective wave from the bottom copper layer is partly reflected at the top air-dielectric interface again with coefficients of ${\tilde{r}}_{21}=r_{21}e^{i\varphi_{21}}$ and partly transmitted with transmission coefficient ${\tilde{t}}_{21}=t_{21}e^{i\theta 21}$, multiple reflections and transmissions within the model have occurred, so the overall reflection is the superposition of the multiple reflections at the two interfaces and expressed as [43]:

(2) $$\tilde{R}=\frac{{\tilde{r}}_{12}-({\tilde{r}}_{12}{\tilde{r}}_{21}-{\tilde{t}}_{12}{\tilde{t}}_{21})e^{i(\theta_{21}-\pi -2\beta d)}}{1-{\tilde{r}}_{21}e^{i(2\beta d+\pi )}}$$

In Equation (2), the angle factor $\theta_{21}-\pi -2\beta d$ represents the phase difference of two nearby reflection waves, *β* = *kd* is the complex propagation phase and *k* is the wave number in the dielectric spacer, *d* is the thickness of dielectric spacer. In order to achieve perfect absorption at resonance frequency, the overall reflection should equal to zero. According to the Equation (2), the amplitude and the phase must satisfy the following two conditions simultaneously for the same frequency:

(3) $$\left|{\tilde{r}}_{12}\right|=\left|{\tilde{r}}_{12}{\tilde{r}}_{21}-{\tilde{t}}_{12}{\tilde{t}}_{21}\right|$$

(4) $$\theta_{21}-\pi -2\beta d=2m\pi ,m=0,\pm 1,\pm 2\cdots$$

**Figure 8:**
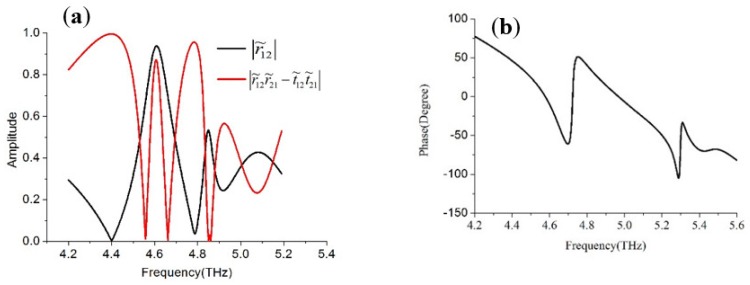
The calculated (**a**) amplitude and (**b**) phase of the reflection and transmission coefficients using the interference theory.

In order to verify whether these two conditions can be satisfied, the reflection and transmission coefficients at individual interfaces are derived from numerical simulations. We plotted the curve of the amplitude condition in Equation (3) and phase condition in Equation (4), respectively, in Figure 8. As shown in Figure 8a, the amplitude of $\left|{\tilde{r}}_{12}\right|$ (in black line) and $\left|{\tilde{r}}_{12}{\tilde{r}}_{21}-{\tilde{t}}_{12}{\tilde{t}}_{21}\right|$ (in red line) cross each other at nearly absorption peak frequency 4.72 and 5.0 THz, where the amplitude condition (3) is satisfied. We can see in Figure 8b, the phase term $\theta_{21}-\pi -2\beta d$ equals to zero at the two absorption frequency, announcing that the phase condition (4) is satisfied too. Thus, both amplitude and phase conditions are satisfied in the proposed design and the ultra-thin dual-band metamaterial absorber can be achieved.

## 4. Conclusions

In summary, we demonstrate the design, characterization, and theoretical interpretations of a terahertz dual-band asymmetric H-shaped perfect metamaterial absorber and PIT-like reflection window between the absorption frequency. The results indicate that the suppressed resonance frequency in the symmetric case of the H-shaped structure can be induced through breaking H-shaped structure in x and y directions simultaneously, and dual-band perfect absorption peak can be achieved. Asymmetry plays an important role in the coupling, and two new plasmon resonance modes appear by splitting the single resonance in the symmetric and interference with each other leading to a PIT-like reflection window. Moreover, a multi-reflection interference theory was discussed as well to offer a distinctive way which allows for getting a physical insight into the absorption mechanism of the metamaterial absorber. However, in addition to the above advantages, there are also limitations in practical application such as polarization and incident angle dependences. The limitations can be solved by integrating the asymmetric structure as a sub-unit into a symmetric structure and the asymmetric structure can be applied in visible light band by scaling down the geometric dimensions to a proper scale in our future work. In a word, the improved designs can be a potential candidate for various applications such as filters, sensors, and some other functional metamaterial devices.
